# Timely Monitoring COVID-19 Vaccine Protection, Berlin, Germany, April 15th to December 15th, 2021

**DOI:** 10.3389/ijph.2022.1604633

**Published:** 2022-03-21

**Authors:** Julia Bitzegeio, Lukas Hemmers, Alexander Bartel, Dirk Werber

**Affiliations:** ^1^ State Office for Health and Social Affairs, Berlin, Germany; ^2^ Department of Infectious Disease Epidemiology, Robert Koch Institute, Berlin, Germany; ^3^ Postgraduate Training for Applied Epidemiology (PAE), Robert Koch Institute, Berlin, Germany; ^4^ European Programme for Intervention Epidemiology Training (EPIET), European Centre for Disease Prevention and Control (ECDC), Stockholm, Sweden; ^5^ Institute for Veterinary Epidemiology and Biostatistics, Freie Universität Berlin, Berlin, Germany

**Keywords:** surveillance, COVID-19, vaccines, vaccine effectiveness, waning immunity, SARS-CoV-2 variants, booster vaccination, vaccine protection

## Introduction

Vaccination is the safest means of protection against coronavirus disease (COVID-19). The level of protection for a population depends on the achieved coverage and the effectiveness of the vaccines. Vaccine effectiveness (VE) against COVID-19 is not constant over time due to the emergence of new SARS-CoV-2 variants [1] and waning of vaccine-derived immunity [2, 3]. Therefore, it is crucial not only to estimate the effectiveness of vaccines against COVID-19, but also to timely monitor changes in their protective effect in the population. We describe a simple approach using surveillance data, based on the screening method, to timely monitor the actual protection of all vaccines applied in the population.

## Data Sources and Definitions

We analyzed data on mandatory notifications of laboratory-confirmed COVID-19 infections in Berlin from April until December 2021 [4]. Laboratory confirmation required detection of severe acute respiratory syndrome coronavirus 2 (SARS-CoV-2) nucleic acid by PCR.

We restricted our analysis to symptomatic cases to reduce possible bias through underdiagnosis of asymptomatic vaccinated cases and to exclude cases that have not yet been investigated by the local public health authority (LPHA). A symptomatic case was defined as a notified person with laboratory-confirmed COVID-19 infection for whom the LPHA has entered at least one symptom compatible with COVID-19 [5], or an onset of disease in the notification software.

A vaccine breakthrough infection (henceforth: a fully vaccinated case) was defined as laboratory-confirmed COVID-19 infection in a person with onset of disease ≥14 days after completion of a full vaccination cycle with any of the vaccines approved in Germany (see Supplementary Material). If the date of onset was missing, we used the date of laboratory confirmation or, if also missing, the notification date.

Booster breakthrough infections were defined as infections in fully vaccinated cases with an additional third dose of Comirnaty or Spikevax.

As of 15 December, 23,079 breakthrough infections have been detected, of which 236 have been detected in persons who had received a booster vaccination. In total, 19,467 had a symptomatic infection, 292 were admitted to hospital, and 87 died (Table 1).

**TABLE 1:** Overview of vaccinations and breakthrough infections in Berlin from January to December 2021 by vaccine as of 15 December 2021. (Berlin, Germany, 2021).

Vaccine	Fully vaccinated population	Breakthrough infections	Median age (IQR)	Symptomatic	Median age sympt. (IQR)	Hospitalized	Dead
Comirnaty	1963346	15710	41 (32–55)	13260	41 (32–54)	230	64
Vaxzevria/Comirnaty	—	1752	37 (30–49)	1485	37 (30–49)	2	1
Spikevax	338710	1636	38 (31–49)	1335	38 (31–48)	12	5
Vaxzevria/Spikevax	—	403	41 (32–52)	346	40 (32–51)	1	0
Vaxzevria	124551	1406	52 (35–63)	1189	52 (35–62)	13	4
Janssen vaccine	121462	1936	35 (28–42)	1726	35 (28–44)	22	3
Comirnaty booster	539916^*^	231	76 (54–83)	125	71 (46–83)	12	10
Spikevax booster	103196^*^	5	76 (30–82)	1	30	0	0
Total	2548069	23079	41 (31–55)	19467	40 (31–53)	292	87

The median ages of cases and symptomatic cases are provided with inter quartile range limits (IQR) to describe their age distribution. *Not included in total.

## Calculation of 14-Day Notification Rates and Relative Risks According to Vaccination Status and Vaccine Protection

**FIGURE 1:**
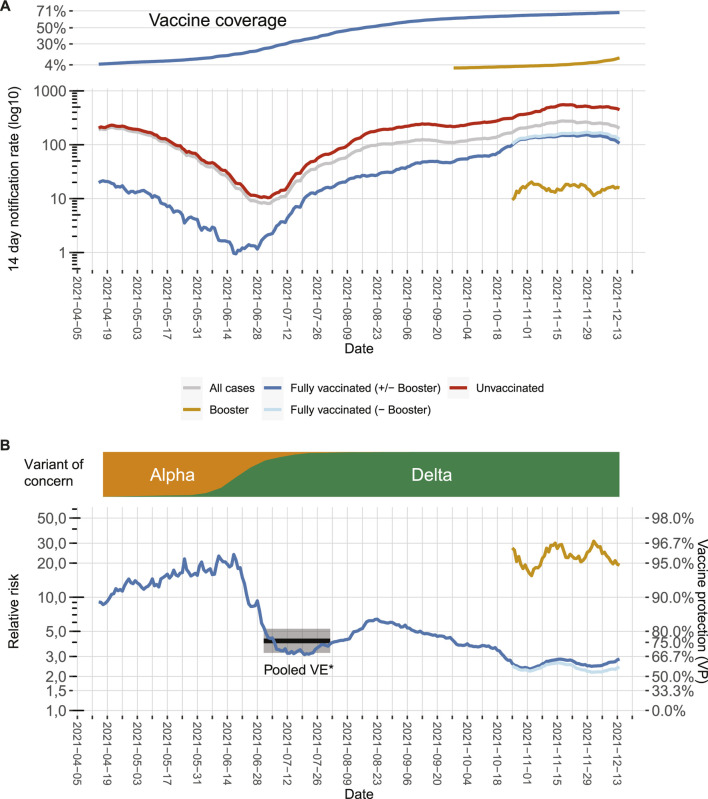
7-day notification rates according to vaccination status, relative risks, and vaccine effectiveness in Berlin from April to December 2021 as of 15 December 2021. **(A)** 7-day notification rates of symptomatic COVID-19 cases over time for the whole population (grey line) or differentiated between the fully vaccinated (dark blue line), fully vaccinated excluding cases with booster vaccinations (light blue line), cases with booster vaccinations (green line), and the unvaccinated population (red line). Vaccination rates of the population in the same time frame are depicted on the top. **(B)** Relative risk (RR) for unvaccinated cases compared to fully vaccinated cases which can be transformed to vaccine protection of overall vaccination (VP; secondary axis) over time (blue line). Likewise, the green line represents the comparison between unvaccinated cases and cases who received a booster vaccination, and the light blue line represents the comparison between unvaccinated cases and fully vaccinated cases, which did not receive a booster vaccination. Distribution of main virus variants over time is depicted on the top. The black line and grey shaded area represent the VE point estimate and the 95% confidence interval calculated for symptomatic infection with the Delta variant in the meta-analysis by Harder et al. [8]. All of the included studies were performed in a phase where the variant of concern Delta was emerging, thus the time period was marked where Delta was emerging in Germany (Berlin, Germany, 2021).

We calculated 14-day notification rates by dividing for each day all symptomatic cases of the last 14 days by the respective population of Berlin, thus creating a 14-day rolling time series of notification rates (Figure 1A). As population for the fully vaccinated cases (*n*_*fullvac*_), we used those who received their second (in case of Janssen, first) vaccination 21 days prior to the notification date [6], as this represented the number of fully vaccinated people at the midpoint of the 14-day rolling window. The population (pop) for the cases with a booster vaccination (n_booster_) was defined as those who received their third vaccination 21 days prior to the notification date. For the 14-day notification rate of unvaccinated cases (n_*unvac*_), we excluded everyone with at least one dose of a vaccine (*n*_*vac*_) and additionally those who recovered in the past 6 months (*n*_*inf6mo*_) from the population of Berlin [7].

$$n_{unvac}\left(t\right)=\;pop\;-\;n_{vac}\left(t-21\right)\;-n_{inf6mo}\left(t\right)$$

To express differences in notification rates according to vaccination status, we divided the 14-day notification rate of unvaccinated cases by the 14-day notification rate of 1) fully vaccinated cases (including those with booster vaccinations), 2) fully vaccinated cases but without booster vaccinations, and 3) all cases that received a booster vaccination, i.e., we computed for each day *t* relative risks *RR*(*t*) for the unvaccinated (formula for case 1, additional formulas can be found in the supplement).

$$RR\left(t\right)\;=\frac{\sum_{i\;=\;t-13}^{t}cases_{unvac}\left(i\right)/n_{unvac}\left(t\right)}{\sum_{i\;=\;t-13}^{t}cases_{fullvac}\left(i\right)/n_{fullvac}\left(t-21\right)}$$

To estimate the protective effect (henceforth: vaccine protection (VP)) against symptomatic infection (Figure 1B), we used: $\;VP\left(t\right)=\left(1-\;\frac{1}{RR\left(t\right)}\right)\times 100$ , analogous to the computation of vaccine effectiveness. Because our estimate reflects an actual snapshot of the combined effectiveness of all vaccines applied in the population, unadjusted for potential confounders (e.g., age), we coined it VP to distinguish it from VE (which usually applies for an adjusted estimate of a single vaccine). Its value lies primarily in observing changes over time, not in accurately estimating VE once.

**FIGURE 2:**
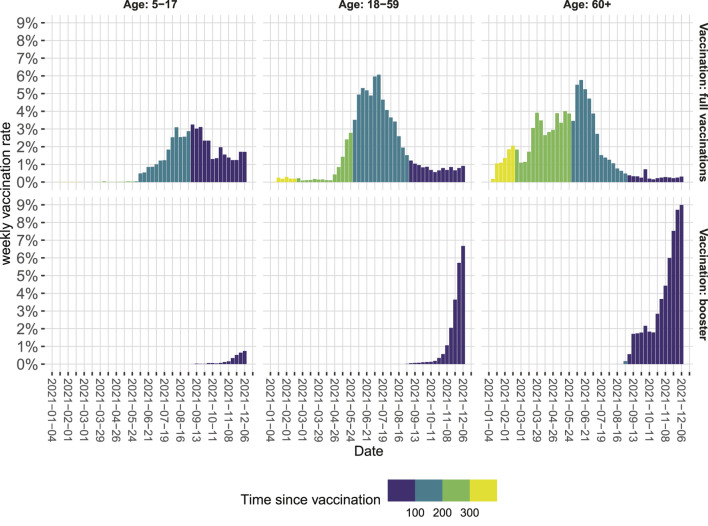
Weekly vaccination rates in Berlin relative to the population in the respective age category from January to December 2021 as of 15 December 2021. Colors indicate the time since vaccination in days (Berlin, Germany, 2021).

By doing so, we made the following observations: First, there is a substantial drop in VP in June, which coincides with the emergence of the SARS-CoV-2 variant of concern (VOC) Delta. The correponding VPs are in line with VE estimates during the emergence of Delta in other countries [1, 8, 9]. Second, for both the time in which VOC Alpha dominated and for the first months in which VOC Delta dominated, there is a moderate increase in VP, which might result from the decreasing average age of the vaccinated population. Vaccines were given first to those with the highest risk of developing severe COVID-19 particularly the older population [10], this might have led initially to an underestimation of VP [11]. Third, since mid-August, the VP constantly decreased from around 84% in August to around 60% in November. This probably signals waning immunity [9, 12, 13]. Time since vaccination is systematically longer for older age groups (Figure 2), potentially increasing the effect of waning immunity visible in our data. Fourth, additional booster vaccinations markedly increased overall VP. When calculating VP only for those who received a booster vaccination, VP increased to around or even higher than the protective level initially observed when VOC Alpha was dominant.

Our estimates apply for the combined effect of all vaccines given to the Berlin population (Table 1) and might be affected by changes over time in the individual contribution of the vaccines. However, when restricting our analysis to the predominantly applied vaccine in Berlin, Comirnaty, we saw the same pattern (data not shown). In general, VP estimates derived from surveillance data may suffer from several biases. Most notably, they are incomplete. Completeness of COVID-19 cases might differ by vaccination status, due to differential health-seeking behavior. Additionally, the accuracy of vaccine coverage data might be limited. Notwithstanding inherent limitations, our VP estimates are congruent to published VE estimates. Notably, interpretation of our analysis should focus on changes over time rather than single-point estimates. As such they can complement more accurate studies on vaccine effectiveness by monitoring VP in the population over time, provided that biases in the surveillance data remain stable over time.

### Conclusion

We were able to detect a remarkable decline in VP corresponding to the introduction of the Delta variant, a gradual decline, likely indicating waning of vaccine-derived immunity and most recently a substantial increase through booster vaccinations. Thus, surveillance data can provide timely insights on the success of COVID-19 vaccination programs for public health authorities and policy makers in upcoming months particularly in the light of new virus variants emerging.

## Abbreviations

COVID-19, coronavirus disease 2019; VE, vaccine effectiveness; SARS-CoV-2, severe acute respiratory syndrome coronavirus 2; LPHA, local public health authority; IQR, inter quartile range; VOC, variant of concern; RR, relative risk; VP, vaccine protection; pop, population.
