# Supplementary material for: Timely Monitoring COVID-19 Vaccine Protection, Berlin, Germany, April 15th to December 15th, 2021
Source: Int J Public Health. 2022 Mar 21;67:1604633. doi: 10.3389/ijph.2022.1604633 (PMC8977412; doi:10.3389/ijph.2022.1604633)
Supplement: Supplementary file 1 [file DataSheet1.PDF]

Calculation of 14-day notification rates and relative risks according to vaccination status and vaccine protection

We calculated 14 day notification rates by dividing for each day all symptomatic cases of the last fourteen days by the respective population of Berlin, thus creating a 14-day rolling time series of notification rates. As population for the fully vaccinated cases ($n_{fullvac}$), we used those who received their second (in case of Janssen first) vaccination 21 days prior to the notification date, as this represented the number of fully vaccinated at the midpoint of the 14-day rolling window. The population for the cases with a booster vaccination ($n_{booster}$) was defined as those who received their third vaccination 21 days prior to the notification date. For the 14-day notification rate of unvaccinated cases (n_{unvac}), we excluded everyone with at least one dose of a vaccine (n_{vac}) and additionally those who recovered in the past six month (n_{inf6mo}) from the population of Berlin.

$$n_{unvac}(t) = pop - n_{vac}(t-21) - n_{inf6mo}(t)$$

To express differences in notification rates according to vaccination status, we divided the 14-day notification rate of unvaccinated cases by the 14-day notification rate of a) fully vaccinated cases (including those with booster vaccination), b) fully vaccinated cases but without booster vaccination and c) all cases that received a booster vaccination, for each day, i.e. we computed a relative risk *RR(t)* due to vaccination for each day *t.*

a)

$$\mathrm{RR}(t) = \frac{\sum_{i=t-13}^{t} \mathrm{cases}_{unvac}(\mathrm{i}) / n_{unvac}(t)}{\sum_{i=t-13}^{t} \mathrm{cases}_{fullvac}(\mathrm{i}) / n_{fullvac}(t-21)}$$

b)

$$\mathrm{RR}(t) = \frac{\sum_{i=t-13}^{t} \mathrm{cases}_{unvac}(\mathrm{i}) / n_{unvac}(t)}{\frac{\left(\sum_{i=t-13}^{t} \mathrm{cases}_{fullvac}(\mathrm{i}) - \sum_{i=t-13}^{t} \mathrm{cases}_{booster}(\mathrm{i})\right)}{\left(n_{fullvac}(t-21) - n_{booster}(t-21)\right)}}$$

c)

$$\mathrm{RR}(t) = \frac{\sum_{i=t-13}^{t} \mathrm{cases}_{unvac}(\mathrm{i}) / n_{unvac}(t)}{\sum_{i=t-13}^{t} \mathrm{cases}_{booster}(\mathrm{i}) / n_{booster}(t-21)}$$

To estimate the effectiveness of all vaccines combined (VP) against symptomatic infection we used: $\mathrm{VP}(t) = 1 - \frac{1}{RR(t)}$ *100

COVID-19 Vaccines approved in Germany as of December 15th 2021

Vaccine Name	Manufacturer	Vaccine type	No. of Doses for full vaccination cycle
Comirnaty (BNT162b2)	BioNTech Pfizer, Mainz Germany / New York, United States (US)	mRNA-Vaccine	2
Spikevax (mRNA 1273)	Moderna, Cambridge, US	mRNA-Vaccine	2
Vaxzevria (ChAdOx1 n CoV 19)	AstraZeneca, Cambridge, United Kingdom	Vector-Vaccine	2
Janssen (Ad26.COV2 S)	Janssen Cilag International NV, Beerse, Belgium	Vector-Vaccine	1
